# Editorial: Animal Toxins as Comprehensive Pharmacological Tools to Identify Diverse Ion Channels

**DOI:** 10.3389/fphar.2019.00423

**Published:** 2019-04-24

**Authors:** Yuri Utkin, Alexander Vassilevski, Denis Kudryavtsev, Eivind A. B. Undheim

**Affiliations:** ^1^Laboratory of Molecular Toxinology, Department of Neuroimmunosignaling, Shemyakin-Ovchinnikov Institute of Bioorganic Chemistry, Russian Academy of Sciences, Moscow, Russia; ^2^Laboratory of Molecular Instruments for Neurobiology, Department of Molecular Neurobiology, Shemyakin-Ovchinnikov Institute of Bioorganic Chemistry, Russian Academy of Sciences, Moscow, Russia; ^3^Phystech School of Biological and Medical Physics, Moscow Institute of Physics and Technology (State University), Dolgoprudny, Russia; ^4^Laboratory of Ligand-Receptor Interactions, Department of Neuroimmunosignaling, Shemyakin-Ovchinnikov Institute of Bioorganic Chemistry, Russian Academy of Sciences, Moscow, Russia; ^5^Centre for Advanced Imaging, The University of Queensland, Brisbane, QLD, Australia; ^6^Department of Biosciences, Centre for Ecology and Evolutionary Synthesis, University of Oslo, Oslo, Norway

**Keywords:** animal toxin, ion channel, voltage-gated channel, ligand-gated channel, snake, scorpion, spider

Changes in the electric potential of cell membranes is a ubiquitous mechanism of cell communication. Key proteins involved are ion channels that allow ion flow through the membranes. The conductivity of ion channels is controlled either by changes in the membrane potential or by ligand binding. According to the means of control, ion channels in general can be divided into two main classes: voltage-gated and ligand-gated channels. The number of discovered ion channels is constantly growing. However, identification of individual channels and study of their particular function in complex living organisms is not an easy task. Fortunately, nature has provided a great number of selective binders that target ion channels with high potency and specificity in the form of toxins from animal venoms. The first toxins used for ligand-gated ion channel studies were α-bungarotoxin from the krait *Bungarus multicinctus* and α-cobratoxin (previously called *Naja naja siamensis* toxin 3) from the cobra *Naja kaouthia* (*siamensis*). They were used to isolate and characterize the nicotinic acetylcholine receptor (Changeux et al., 1970; Karlsson et al., 1972). Since then, the number of animal toxins applied for ion channel studies has increased enormously, with discoveries of ion channel-specific toxins driving progress in investigations of their targeted channels. Recent developments in analytical techniques allow identification and isolation of new toxins, which may serve as comprehensive tools for ion channel research. In this Research Topic, reviews and original articles discussing different aspects of toxin discovery and applications for ion channel studies are presented.

The mini-review by Bajaj and Han provides a brief reflection on animal toxins targeted at potassium, sodium, and calcium channels. Among potassium channels, three representative families, i.e., inwardly rectifying (Kir), voltage-gated (K_V_), and calcium-activated (K_Ca_) potassium channels are considered. Several toxins acting on these channels from different animals are briefly discussed. Different types of cone snail, spider and sea anemone toxins are presented as effectors of voltage-gated sodium channels. Ω-Conotoxins and Ω-agatoxins are mentioned as the best-described blockers of voltage-gated calcium channels.

Among voltage-gated sodium channels (Na_V_), one of the most intriguing subtypes from the drug development perspective is Na_V_1.7, which is involved in pain perception. Toxins specifically targeting this subtype of sodium channel are considered in detail in review by Gonçalves et al.. The review highlights Na_V_1.7 as a pharmacological target, and points to several discoveries of spider toxins possessing analgesic properties due to their interactions with Na_V_1.7. The structural features of these toxins that are essential for their interaction with Na_V_1.7 are discussed, and data about *in vitro* activity on DRG neurons are presented for several toxins and their analogs. Overall, the Na_V_1.7-selective spider toxins are exciting leads for therapeutic development.

The complex spatial organization of ion channels requires the application of different approaches for their investigation. Bioinformatics and molecular modeling are widely used for the prediction of those channel properties, which at the present time cannot be established by “wet biochemistry.” Animal toxins play a significant role in such predictions as clearly demonstrated by Tikhonov and Zhorov in their review. The homology models of the sodium channel subtype Na_V_1.4 built using TTX, STX, and μ-conotoxins as molecular probes, are compared with cryo-electron microscopy structures of cockroach sodium channel Na_V_PaS and Na_V_1.4. The comparison shows a good correlation of the predicted and experimentally determined structures, which justifies the employment of toxins and other ion channel ligands as molecular probes.

The abundance of ion channels necessitates a search for new toxins, which may serve as specific and selective tools for particular ion channel investigation. Wang et al. address this demand by presenting new sodium channel inhibitors from leech saliva. They have discovered in salivary glands of the leech *Haemadipsa sylvestris* a new family of peptides, which manifest significant analgesic activity. The peptides comprise 22–25 amino acid residues with two intramolecular disulfide bridges, and are specific inhibitors of sodium channel subtypes Na_V_1.8 and Na_V_1.9. These peptides may be considered as hits to develop analgesics.

Toxins possessing new structural features emerge occasionally. Recently, polypeptide toxins with an unusual tandem repeat organization have been described. These toxins presenting unique pharmacological properties are considered in the article by Maxwell et al., which describes a new bioinformatics pipeline for mining such toxins. A new name SCREPs (Secreted, Cysteine-rich REpeat Peptides) is suggested for these molecules, and the analysis of UniProt by data mining techniques yields a vast diversity of potential tandem-repeat polypeptides. Predicted SCREPs include a structural diversity that goes well beyond previously described SCREPs, such as the two-domain toxins from spider venoms.

Altogether, the publications in this Research Topic issue demonstrate that animal toxins are widely used for ion channel studies and continue to serve as important pharmacological tools for the characterization of diverse ion channels.

## Author Contributions

All authors listed have made a substantial, direct and intellectual contribution to the work, and approved it for publication.

### Conflict of Interest Statement

The authors declare that the research was conducted in the absence of any commercial or financial relationships that could be construed as a potential conflict of interest.
